# Case Report: A patient with the rare third-generation TKI-resistant mutation *EGFR* L718Q who responded to afatinib plus cetuximab combination therapy

**DOI:** 10.3389/fonc.2022.995624

**Published:** 2022-10-31

**Authors:** Guoqing Zhang, Beibei Yan, Yanan Guo, Hang Yang, Xiangnan Li, Jindong Li

**Affiliations:** Department of Thoracic Surgery and Lung Transplantation, First Affiliated Hospital of Zhengzhou University, Zhengzhou, Henan, China

**Keywords:** lung adenocarcinoma, EGFR, drug resistance, L718Q, afatinib, cetuximab

## Abstract

Third-generation tyrosine kinase inhibitors (TKIs), such as osimertinib, almonertinib and furmonertinib, overcome the mechanisms of resistance to first-generation inhibitors (such as gefitinib, erlotinib and icotinib) by incorporating an acrylamide group that alkylates the Cys797 of *EGFR* T790M. However, drug resistance is inevitable, even for third-generation TKIs. Screening for drug-resistant mutations by repeat biopsy and repeat gene sequencing is necessary after TKI treatment. Among various third-generation TKI-resistant mutations, secondary mutation of the L718 residue of *EGFR* exon 18 was found in approximately 8% of patients and is responsible for drug resistance *in vitro* and *in vivo*. Furthermore, there is limited clinical experience of targeted therapy for this mutation. Herein, we report for the first time that afatinib and cetuximab combination therapy can overcome such drug resistance.

## Highlights

1. The *EGFR* L718Q mutation is responsible for resistance to third-generation TKIs *in vitro* and *in vivo*, and few targeted regimens can effectively overcome this resistance.

2. We demonstrated the first use of combination therapy with afatinib and cetuximab to achieve response in a patient with almonertinib-resistant lung adenocarcinoma bearing the L858R/L718Q mutation.

## Patient history

**Figure 1:**
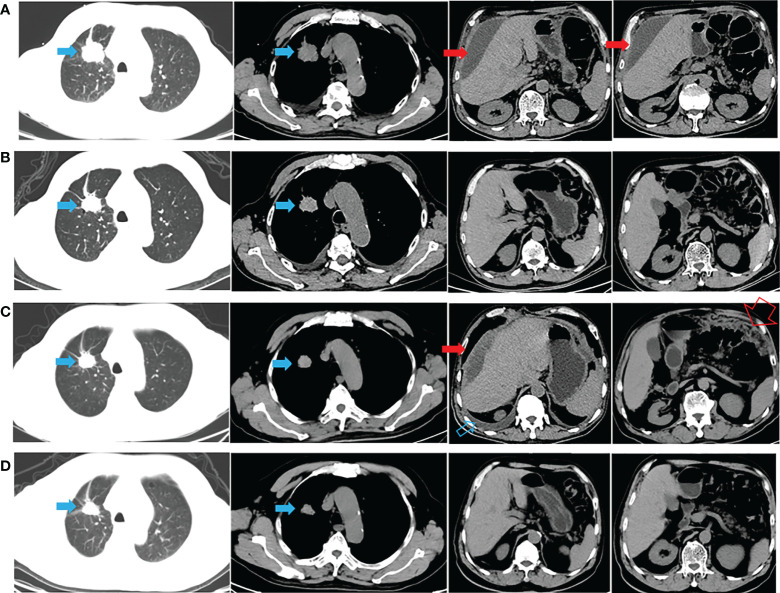
CT appearance across the therapeutic process. **(A)** Thoracoabdominal CT at diagnosis (after thoracoabdominal drainage); **(B)** 1 month after almonertinib treatment; **(C)** hydrothorax and ascites appeared after almonertinib resistance (after thoracoabdominal drainage); **(D)** hydrothorax and ascites disappeared 1 month after afatinib plus cetuximab treatment. The solid blue arrow indicates the primary lesion; the open blue arrow indicates the hydrothorax; the solid red arrow indicates the malignant ascites; the open red arrow indicates the greater omentum metastasis.

**Figure 2:**
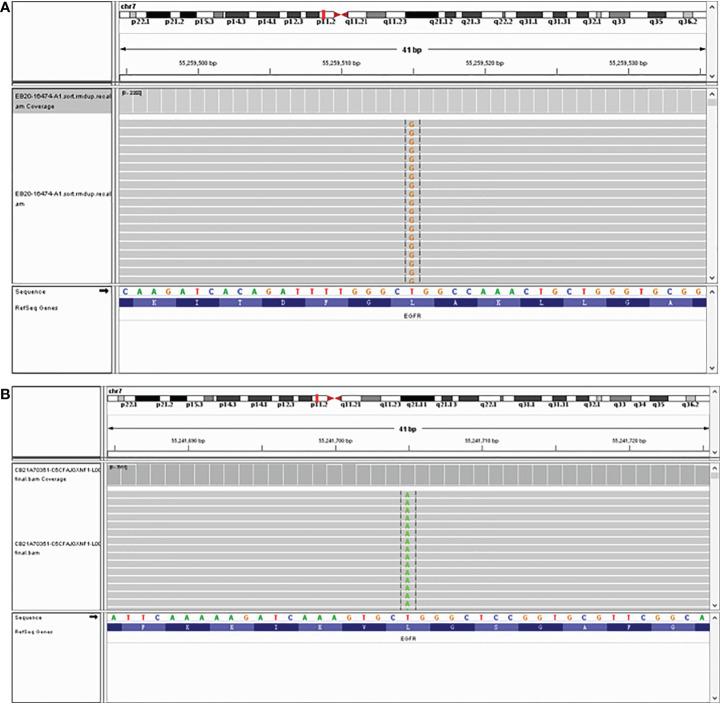
**(A)** Integrative Genomics Viewer snapshot of the L858R mutation in *EGFR* exon 21. **(B)** Integrative Genomics Viewer snapshot of the L718Q mutation in *EGFR* exon 18.

A 72-year-old man with a smoking history of approximately 20-pack-years complained of abdominal distension and was admitted to our hospital on August 22, 2020. Thoracoabdominal computed tomography (CT) (**Figure 1A**) and a radionuclide bone scan showed a 2.8-cm right upper lung mass, hydrothorax, ascites and multiple bone metastases (skull, right clavicle, multiple vertebrae, and pelvis). Left cervical lymph node metastasis was diagnosed based on color Doppler ultrasonography. CT-guided biopsy showed that the mass was lung adenocarcinoma, and the patient was diagnosed with adenocarcinoma of the right upper lung with multiple metastases (cT1bN3M1c, stage IVB). Tissue from the biopsy was analyzed *via* next-generation sequencing (NGS) of a panel of 8 cancer-related genes (Geneseeq, Nanjing, China) on September 8, 2020. Mutations were detected in *EGFR* exon 21 (L858R) (abundance: 14.5%) and *PIK3CA* exon 8 (E474D) (abundance: 36.6%), as shown in **Figure 2A**. The patient received first-line therapy with the third-generation TKI almonertinib (110 mg/d) and bisphosphonates (zoledronic acid, 4 mg, q4w). Stable disease was achieved initially, but the patient ultimately experienced progressive disease (appearance of hydrothorax and ascites), with a progression-free survival (PFS) time of 12 months (**Figures 1B, C**). After disease progression, the patient was admitted to the local hospital and received 2 cycles of chemotherapy with cis-platinum (75 mg/m^2^, d1) and pemetrexed (500 mg/m^2^, d1). However, the hydrothorax and ascites were not controlled. Then, the patient was admitted to our hospital, and metastatic lung adenocarcinoma was diagnosed in the ascites sediment. A repeat examination for gene mutations was performed on the ascites sediment (Geneseeq, Nanjing, China), and mutations in *EGFR* exon 21 (L858R) (abundance: 1.7%), *EGFR* exon 18 (L718Q) (abundance: 0.8%) and *TP53* (V157F) (abundance: 1.6%) were found (**Figure 2B**).

The patient’s case was discussed with a multidisciplinary team (MDT). Importantly, the patient was fully informed of the benefits and risks of the proposed therapy and signed an informed consent form. Studies have shown that afatinib combined with a lower dose of cetuximab (250 mg/m^2^) may have improved tolerability compared to a higher dose of cetuximab (500 mg/m^2^) (1, 2). Thus, combination therapy with afatinib (40 mg daily) and cetuximab (250 mg/m^2^, q2w) was started on November 16, 2021. CT performed after 1 month showed stable lung and metastatic lesions, and the hydrothorax and ascites were well controlled (**Figure 1D**). Then, follow-up was performed every 2-3 months, with the most recent follow-up on June 08, 2022 (**Supplementary Image 1**). A mild gastrointestinal reaction was the only adverse event (AE) observed. The patient is still receiving the combination regimen and has a satisfactory quality of life, with a PFS of nearly 7 months. The overall survival (OS) exceeded 21 months from the initial pathological diagnosis (**Figure 1** and **Supplementary Image 1**).

## Discussion

The six broad resistance mechanisms against third-generation EGFR TKIs among patients harboring *EGFR* mutant non-small cell lung carcinoma (NSCLC) are as follows: loss of T790M, maintenance of T790M, *EGFR* mutations (C797S, G724S, L718Q), bypassing of pathway activation, SCLC transformation and enhancement of autophagy (3, 4). Targeted treatment is currently being developed for each resistance mechanism (5). For NSCLC with the C797S(+)/T790M(+) mutation, cetuximab combined with brigatinib or EAI045 is effective for the cis-C797S/T790M mutation, whereas a first-generation TKI combined with a third-generation TKI is effective for the trans-C797S/T790M mutation. For NSCLC with the C797S(+)/T790M(-) mutation, first- and second-generation EGFR TKIs are effective. For the other resistance mechanisms, promising targeted therapy combinations are emerging (third-generation EGFR TKIs combined with MEK inhibitors, MET inhibitors and HER2 inhibitors); other fourth-generation EGFR TKIs (BLU-945, TQB3804, etc.) are also being explored.

The *EGFR* T790M mutation is responsible for the reduction in or even lack of inhibitory potency of first-generation inhibitors (6). *In silico* protein structure modeling suggests that this effect may result from changes in the steric and lipophilic properties of the gatekeeper residue (7). Similarly, targeted therapy using third-generation inhibitors is rendered nonfunctional by the L718Q mutation due to a remarkable increase in spatial conflict and a decrease in local hydrophobicity (8).

Preclinical studies have shown that irreversible EGFR TKIs demonstrated activity against T790M (9, 10), but these findings failed to translate into clinically significant results (11). Similar to the case for the L718Q mutation, there are also inconsistencies between studies. A preclinical study showed that cells containing Del19/L718Q or L858R/L718Q retain sensitivity to first- and second-generation inhibitors, such as gefitinib and afatinib. However, strong resistance to osimertinib was observed (8, 12). The *EGFR* tertiary mutations Del19/L718Q/T790M and L858R/L718Q/T790M were resistant to first-, second- and third-generation TKIs (8, 12) (**Table 1**). Interestingly, Yang (8) demonstrated that cells with the Del19/L718Q/T790M mutations showed slightly less resistance than those with the L858R/L718Q/T790M mutations (8). However, another study failed to replicate this result (12).

**Table 1:** Preclinical data evaluating the sensitivity of EGFR TKIs in cell models harboring the L718Q mutation.

Author and year	Study object	Mutation type	IC_50_ value (nmol/L)		
			First-generation^&^	Second-generation^$^	Third-generation^#^
Ercan 2015 (12)	Ba/F3 cell lines	L718Q	513	2.76	3080
		ΔE746_A750/L718Q	61	2	1178
		L858R/L718Q	1117	7.94	2299
		ΔE746_A750/T790M/L718Q	>3300	2115	2547
		L858R/T790M/L718Q	>3300	1209	1977
Yang 2018 (8)	Ba/F3 cell lines	cis-Ex19del/L718Q*	NA		NA
		cis-L858R/L718Q	≈500		>1000
		cis-Ex19del/T790M/L718Q	>1000		>500
		cis-L858R/T790M/L718Q	>1000		>1000

*cells with this type of mutation could not grow upon IL3 withdrawal.

^&^gefitinib.

^$^afatinib.

^#^including WZ4002 and osimertinib. NA, not applicable.

In terms of clinical studies, most of the mutations studied to date are *EGFR* L858R/L718Q (13–17) mutations, with only two *EGFR* tertiary mutations, Del19/L718Q/T790M (18) and L858R/L718Q/T790M (19), reported (**Table 2**). Studies suggest that patients with complex secondary L718Q mutations [*EGFR* L858R/L718Q (13–15, 17) or *EGFR* Del19/L718Q/T790M (18)] experience poor therapeutic response to targeted therapy, with PFS of 1 month for first-generation treatment and 4-6 months for second-generation treatment. Interestingly, Shen reported that a patient with an *EGFR* Del19/L718Q/T790M mutation who acquired resistance to osimertinib was sensitive to treatment with almonertinib (18). This was surprising because the third-generation TKIs almonertinib and osimertinib share a similar mechanism for EGFR inhibition and drug resistance. Upon careful reading of the study, we found that osimertinib was discontinued due to AEs, not drug resistance, as described in the title. In this study (18), almonertinib inhibited the growth of a lesion with an *EGFR* Del19/L718Q/T790M mutation, with a PFS of 4 months, which was not consistent with the results of preclinical studies (8, 12). The sensitivity of the *EGFR* Del19/L718Q/T790M mutation to almonertinib may reflect the complex responses of complex mutations; it is also possible that the L718Q mutation does not always confer resistance to almonertinib.

**Table 2:** Reported cases of lung adenocarcinoma with the L718Q mutation.

Year and author	Pathology	Sex	age	Smoking status	Primitive mutation	Previous targeted therapy	Acquired mutations	Treatment	Treatment response	PFS	OS
2016 Bersanelli (19)	LAC	Female	71 y	NR	L858R	Gefitinib, osimertinib	EGFR L718Q EGFR T790M	NR	NR	NR	NR
2019 Liu (13)	LAC	Female	65 y	Nonsmoker	L858R	Icotinib, osimertinib	EGFR L718Q EGFR Amp BRAF G466R	Afatinib	PR	4 m	23 m
2019 Ma (15)	LAC	Male	55 y	NR	L858R	Gefitinib, osimertinib	EGFR L718Q TP53	Icotinib	SD	1 m	NR
2020 Song (16)	LAC	Male	44 y	Nonsmoker	L858R	Icotinib, osimertinib	EGFR L718Q EGFR Amp	Cisplatin/gemcitabine	PR	5 m	30 m
2020 Yang (17)	LAC	Female	69 y	Nonsmoker	L858R	Gefitinib, osimertinib	EGFR L718Q	Afatinib	PR	4 m	NR
2021 Shen (18)	LAC	Female	45 y	NR	Del 19	Icotinib	EGFR L718Q PLEKHH2-ALK fusions EGFR T790M	Almonertinib	PR	4 m	38 m
2021 Shen (14)	LAC	Female	64 y	Nonsmoker	L858R	Gefitinib, osimertinib	EGFR L718Q	Dacomitinib	PR	6 m	NR
2022 Zhang (our case)	LAC	Male	73 y	Smoker	L858R	Almonertinib	EGFR L718Q TP53	Afatinib+cetuximab	PR	≈7 m	>21 m

LAC, lung adenocarcinoma; NR, not reported; y, year; PR, partial response; SD, stable disease.

Second-generation inhibitors, such as afatinib, may play an important role in the treatment of disease that is resistant to first/third-generation inhibitors, which implies that second-generation inhibitors have some unique properties. For example, afatinib is theoretically a multitarget inhibitor that can irreversibly bind to Cys797 of EGFR, Cys805 of HER2 and Cys803 of HER4 (20). Furthermore, the acrylamide warhead of afatinib alkylates Cys797, thus circumventing ATP competition and overcoming drug resistance caused by T790M or L718Q mutations (21). According to preclinical studies, Del19/L718Q and L858R/L718Q mutations exhibited good sensitivity to second-generation TKIs such as afatinib; this was further confirmed in clinical cases, as patients with *EGFR* L858R/L718Q mutations achieved 4- to 6-month PFS under these treatments.

EGFR TKIs and EGFR monoclonal antibodies (mAbs) have been combined to increase the intensity of EGFR inhibition because they can exert synergistic antitumor effects and because their combination permits a reduced dose of each drug to be used, allowing AEs to be controlled without impairing the therapeutic effect. In our previous study, we reviewed in detail the potential mechanisms (such as direct inhibition, receptor internalization, and immunological effects) by which cetuximab could increase the efficacy of EGFR TKIs (22). This combination regimen may be particularly effective for *EGFR* exon 20 ins mutations (1), C797S/T790M/sensitive mutations (23), and *EGFR*–intergenic region (*IGR*) (*SEC61G*) fusion/*EGFR* amplification mutations (24). We searched databases including PubMed, Embase, OvidSP, and Web of Science up to July 2022 and found no investigation focused on clinical combination therapy for the L718Q mutation; therefore, there is no clearly preferred therapeutic option. We predicted that the patient might benefit from a second-generation EGFR TKI and EGFR mAb combination because of the L858R/L718Q mutation.

A major feature of this case was that the primary lesion remained stable throughout the treatment. Chest discomfort and abdominal distension were the chief complaints after the progression of disease. Correspondingly, hydrothorax and ascites were the major manifestations of almonertinib resistance, and the control of hydrothorax and ascites was evidence that this combination therapy works. In addition to the underlying theory presented above, the occurrence of *TP53* mutation might have conferred almonertinib resistance in this specimen, and the loss of *PIK3CA* would be a potential mechanism for sensitization to afatinib plus cetuximab. On the other hand, the NGS results were obtained from ascites sediment; thus, it is possible that the primary lung tumor and hydrothorax/ascites had mixed responses to treatment due to tumor heterogeneity. Gene mutation examination for primary lung tumors, when necessary, may provide more information. Finally, other modes of combination therapy (such as TKI combined with antiangiogenic therapy and/or chemotherapy) and other drug delivery routes (such as intraluminal cetuximab perfusion combined with oral afatinib) after ascites and pleural effusion progression may also be interesting and worthy of further exploration.

## Conclusion

Herein, we demonstrated the first use of combination therapy with afatinib and cetuximab for a patient with an L858R/L718Q mutation who achieved a response after almonertinib resistance. However, a limitation of our study is that our patient could have responded by chance because of limited data, even though a series of theoretical foundations for the response exists. In the future, we intend to pursue fundamental research on such rare mutations and explore their impacts on the response to combination therapy.

## Data availability statement

The datasets presented in this article are not readily available because further studies are in progress about this case. Requests to access the datasets should be directed to the corresponding author.

## Ethics statement

The studies involving human participants were reviewed and approved by Zhengzhou University Institutional Review Board. The patients/participants provided their written informed consent to participate in this study.

## Author contributions

GZ, BY, and YG contributed to conception and design of the study. GZ wrote the first draft of the manuscript. All authors contributed to manuscript revision, read, and approved the submitted version.

## Funding

This study was supported by the Key Scientific Research Projects of Institutions of Higher Learning in Henan Province (No. 21A320032).

## Conflict of interest

The authors declare that the research was conducted in the absence of any commercial or financial relationships that could be construed as a potential conflict of interest.

## Publisher’s note

All claims expressed in this article are solely those of the authors and do not necessarily represent those of their affiliated organizations, or those of the publisher, the editors and the reviewers. Any product that may be evaluated in this article, or claim that may be made by its manufacturer, is not guaranteed or endorsed by the publisher.

## Supplementary material

Supplementary Image 1 Timeline of patient events from admission until this report. AC, adenocarcinoma; OS, overall survival; PD, progression of disease; PFS, progression-free survival.

## Abbreviations

AE, adverse event; EGFR, epidermal growth factor receptor; mAb, monoclonal antibody; MDT, multidisciplinary team; NGS, next-generation sequencing; OS, overall survival; PD, progression of disease; PFS, progression free survival; TKI, tyrosine kinase inhibitor.
