# Supplementary figures and images for: Case Report: A patient with the rare third-generation TKI-resistant mutation EGFR L718Q who responded to afatinib plus cetuximab combination therapy
Source: Front Oncol. 2022 Oct 31;12:995624. doi: 10.3389/fonc.2022.995624 (PMC9659857; doi:10.3389/fonc.2022.995624)

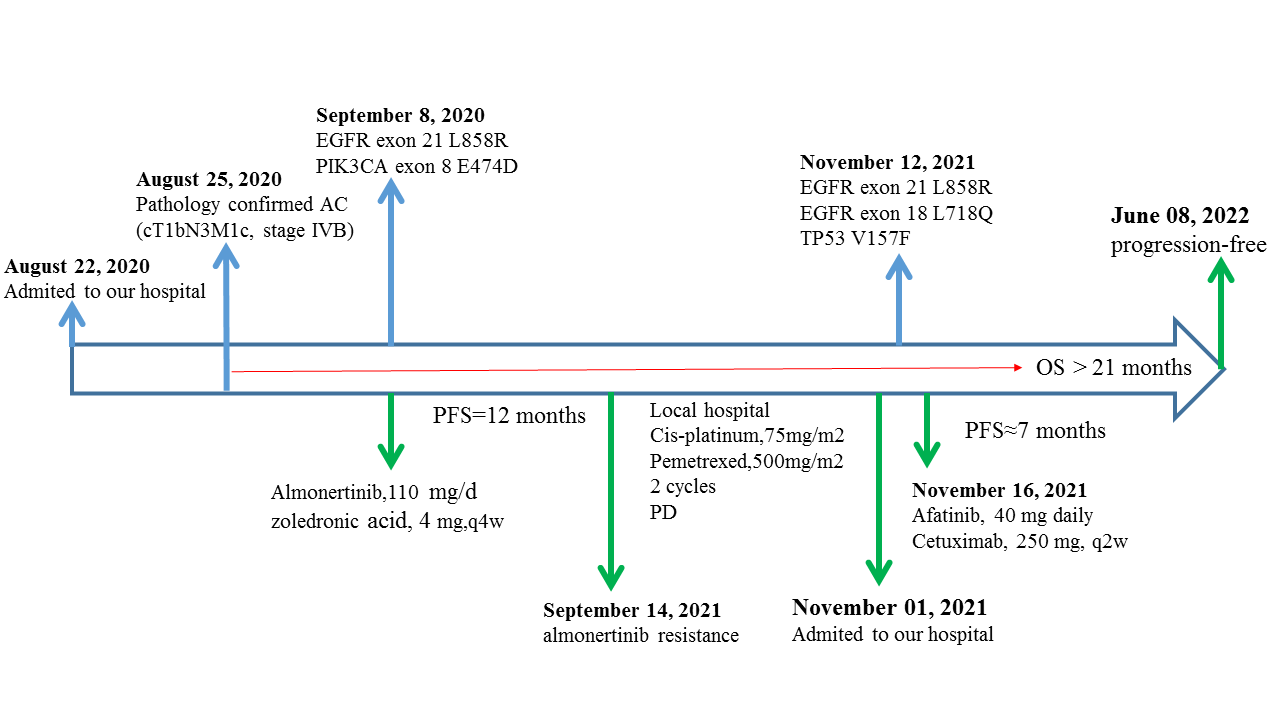

Supplement: Supplementary Image 1 — Timeline of patient events from admission until this report. AC, adenocarcinoma; OS, overall survival; PD, progression of disease; PFS, progression-free survival. [file Image_1.tif]
